# *Serratia marcescens* in Intensive Care Units: Molecular Epidemiology, Biofilm-Mediated Persistence, Antimicrobial Resistance, and Genomic Surveillance

**DOI:** 10.3390/ijms27135697

**Published:** 2026-06-24

**Authors:** Tao-An Chen, Ya-Ting Chuang, Hua-Yu Lin, Ya-Fung Chang, Yu-Ho Hsieh, Cheng-Hsien Chen, Chang-Sheng Lin, Yi-Jen Wang

**Affiliations:** 1 Division of Respiratory Therapy, Department of Chest Medicine, Show Chwan Memorial Hospital, Changhua City 50009, Taiwan; b117100045@tmu.edu.tw; 2 Department of Nursing, Show Chwan Memorial Hospital, Changhua City 50009, Taiwan; s09901383@cjc.edu.tw (Y.-T.C.);; 3 Department of Surgery, Show Chwan Memorial Hospital, Changhua City 50009, Taiwan; 4 Department of Internal Medicine, Show Chwan Memorial Hospital, Changhua City 50009, Taiwan; 5 Department of Infectious Diseases, Show Chwan Memorial Hospital, Changhua City 50009, Taiwan

**Keywords:** *Serratia marcescens*, intensive care unit, nosocomial infection, biofilm, antimicrobial resistance, environmental reservoir, ventilator-associated transmission, infection control, genomic surveillance, water systems

## Abstract

*Serratia marcescens* has emerged as an important opportunistic pathogen in intensive care units (ICUs), where critically ill patients, invasive devices, antimicrobial exposure, and complex environmental reservoirs create favorable conditions for colonization, infection, and recurrent outbreaks. This narrative review synthesizes evidence from the past decade regarding the clinical and molecular epidemiology, environmental persistence, device-associated transmission, biofilm-mediated resistance, and infection-control strategies of *S. marcescens* in ICU settings. The literature was reviewed using an integrative approach informed by Ferrari’s narrative review framework, with thematic synthesis across clinical, microbiological, environmental, and genomic domains. Recent evidence indicates that ICU-associated *S. marcescens* infections frequently involve respiratory tract colonization, ventilator-associated pneumonia, bloodstream infection, urinary tract infection, and device-related transmission. Hospital water systems, sink drains, wet surfaces, ventilator circuits, reusable equipment, and contaminated antiseptic or liquid products may serve as persistent reservoirs, particularly when biofilm formation supports long-term survival and recurrent dissemination. At the molecular level, *S. marcescens* demonstrates substantial genomic diversity, intrinsic and acquired antimicrobial resistance, inducible AmpC β-lactamase activity, efflux-mediated tolerance, and plasmid-associated resistance gene transfer. This review particularly emphasizes the molecular determinants that enable *S. marcescens* to persist in ICU ecosystems, including AmpC-mediated β-lactam resistance, efflux-associated tolerance, quorum-sensing-regulated biofilm formation, plasmid-mediated horizontal gene transfer, and WGS-defined clonal transmission. Whole-genome sequencing, rapid molecular diagnostics, active surveillance, environmental sampling, and integrated infection-control bundles have become increasingly important for distinguishing clonal outbreaks from endemic transmission and guiding timely interventions. Emerging perspectives emphasize the need to combine antimicrobial stewardship, environmental engineering, respiratory-care auditing, anti-biofilm strategies, and AI-assisted real-time surveillance into adaptive ICU infection-control frameworks. Overall, *S. marcescens* should be regarded not merely as an episodic outbreak organism, but as a highly adaptable ICU-associated pathogen requiring multidisciplinary prevention strategies.

## 1. Introduction

*Serratia marcescens* is a Gram-negative, facultatively anaerobic bacillus belonging to the order *Enterobacterales* and is widely recognized as an opportunistic pathogen associated with healthcare-associated infections (HAIs) [1,2,3]. Within the genus *Serratia*, *S. marcescens* is considered the most clinically significant species [3,4,5,6]. Historically regarded as a non-pathogenic environmental saprophyte, it has gradually emerged as an important cause of severe infections, particularly among hospitalized, immunocompromised, and critically ill patients [4,5,6,7].

A defining feature of *S. marcescens* is its remarkable ecological adaptability [8,9,10,11]. The organism is widely distributed in natural and healthcare environments, including soil, water systems, hospital surfaces, medical devices, and moist reservoirs, which facilitates persistent colonization and healthcare-associated transmission [8,9,10,11]. Clinically, *S. marcescens* has been implicated in a broad spectrum of infections, including respiratory tract infections, urinary tract infections, bloodstream infections, meningitis, surgical site infections, and device-associated infections [12,13,14,15,16,17,18]. These infections are particularly relevant in patients exposed to invasive procedures, prolonged hospitalization, antimicrobial therapy, and intensive care support [12,13,14,15,16,17,18].

In intensive care units (ICUs), *S. marcescens* represents a significant nosocomial pathogen because of its ability to colonize respiratory equipment, vascular catheters, water reservoirs, sinks, and other environmental niches [2,7,10,11,19]. These characteristics contribute to endemic persistence, recurrent transmission, and outbreak potential in high-risk clinical settings. Outbreaks in neonatal intensive care units (NICUs) have been especially well documented and are frequently associated with substantial morbidity and mortality, underscoring the clinical severity of *S. marcescens* infections in vulnerable populations [9,15,20,21,22,23].

Recent advances in microbial genomics have further expanded the understanding of *S. marcescens* biology and epidemiology. From a taxonomic and microbiological perspective, *S. marcescens* is now recognized as part of a broader *S. marcescens* complex that includes several closely related species, such as *S. bockelmannii*, *S. nematodiphila*, and *S. ureilytica* [24,25]. This increasing taxonomic complexity has important implications for species identification, molecular epidemiology, outbreak investigation, and genomic surveillance.

Antimicrobial resistance is another major concern in *S. marcescens* infections. The organism exhibits both intrinsic and acquired resistance mechanisms, including chromosomally encoded AmpC β-lactamases, which can hydrolyze a wide range of β-lactam antibiotics, including cephalosporins and β-lactam/β-lactamase inhibitor combinations [26,27,28]. AmpC expression may be inducible or derepressed through regulatory mutations, and resistance determinants may also be mobilized through plasmids, thereby contributing to therapeutic failure and complicating antimicrobial selection [26,29,30]. In addition, *S. marcescens* is intrinsically resistant to polymyxins and has demonstrated the ability to acquire carbapenem resistance genes, further limiting treatment options for severe infections [31,32]. The global rise of carbapenem-resistant Enterobacterales, particularly during and after the COVID-19 pandemic, has intensified concerns regarding multidrug-resistant *S. marcescens* in healthcare settings [31,33,34].

**Figure 1:**
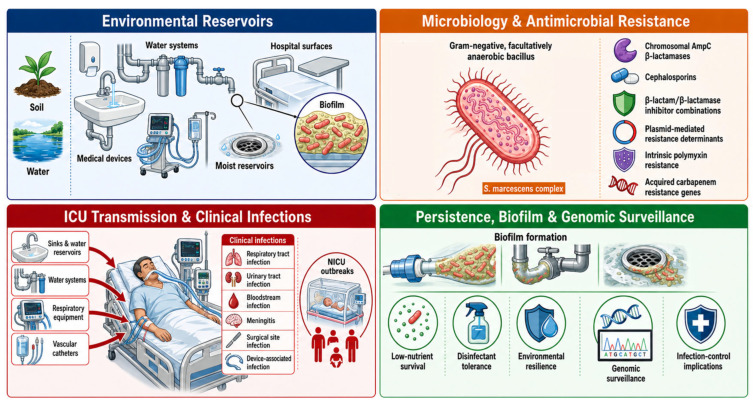
Conceptual Overview of Clinical Transmission, Environmental Persistence, Antimicrobial Resistance, Biofilm Formation, and Genomic Surveillance of *Serratia marcescens* in ICU Settings. Created with BioRender.com (confirmation number: 6a326a7416a9a09fa3d6aa78).

Beyond antimicrobial resistance, *S. marcescens* possesses multiple virulence and survival determinants that facilitate persistence in hospital ecosystems. These include survival in low-nutrient environments, tolerance to disinfectants, environmental resilience, and biofilm formation on medical devices and water-associated surfaces [3,35]. Biofilm-associated growth enhances antimicrobial tolerance, supports long-term environmental survival, and promotes recurrent contamination of hospital reservoirs. Together, these features make *S. marcescens* difficult to eradicate and contribute to prolonged or recurrent outbreaks in ICU environments [2,35]. To provide a conceptual overview of these interconnected issues, Figure 1 summarizes the multifaceted clinical and environmental challenges of *S. marcescens* in ICU environments, including environmental reservoirs, healthcare-associated transmission pathways, antimicrobial resistance mechanisms, biofilm-mediated persistence, and the role of genomic surveillance in outbreak investigation and infection-control strategies.

Despite growing recognition of its clinical and epidemiological importance, the available literature on ICU-associated *S. marcescens* remains fragmented. Many studies are limited to case reports, single-center outbreak investigations, or retrospective observational analyses, and few reviews have comprehensively integrated its molecular epidemiology, environmental persistence, biofilm-mediated resistance, antimicrobial resistance, and genomic surveillance strategies in ICU settings [1,2,5,6,18]. Unlike prior reviews that primarily focus on clinical outbreaks or infection-control measures, this review frames ICU-associated *S. marcescens* through a molecular and genomic lens. We specifically integrate evidence on clonal diversity, antimicrobial resistance determinants, plasmid-mediated gene transfer, quorum-sensing-regulated biofilm formation, environmental adaptation, and WGS-based surveillance to clarify how molecular mechanisms shape persistence, transmission, and outbreak control in ICU ecosystems.

## 2. Methods

We conducted a literature search using a methodology informed by Ferrari’s narrative review framework [36]. This review provides an integrative overview of the molecular epidemiology, virulence determinants, antimicrobial resistance (AMR), and clinical relevance of *S. marcescens* in ICU settings over the past decade. Prior to formal synthesis, T.-A.C., Y.-T.C., and Y.-J.W. performed preliminary scoping searches to refine key concepts and optimize search terms. Subsequently, structured literature searches were conducted across PubMed/MEDLINE, EMBASE, and Google Scholar for studies published between January 2016 and March 2026. Although search strategies were tailored to each database, a core Boolean framework was consistently applied: (“*Serratia marcescens*” [Title/Abstract] OR “*S. marcescens*” [Title/Abstract]) AND (“Intensive Care Units” [Mesh] OR ICU OR “intensive care” OR “critical care”). Additional keywords related to AMR, virulence factors, epidemiology, and nosocomial infections were incorporated where appropriate to enhance retrieval sensitivity, and filters were applied to limit results to studies published within the past 10 years and, when feasible, to full-text articles. In addition to peer-reviewed literature, grey literature sources—including reports and surveillance documents from infectious disease and critical care organizations—were manually searched, and reference lists of relevant articles were screened to identify further studies. All identified records were independently reviewed by T.-A.C., Y.-T.C., and Y.-J.W., and studies were selected based on their relevance to ICU-associated *S. marcescens*. The included literature was then collectively synthesized into thematic domains. As this study was conducted as a narrative review rather than a systematic review, a PRISMA flow diagram was not included. The reviewed literature was primarily limited to studies published in English. Any differences in study selection were resolved through discussion and final consensus among T.-A.C., Y.-T.C., and Y.-J.W. After this consensus-based evaluation, a total of 67 articles were included and collectively synthesized into thematic domains.

## 3. Molecular Epidemiology and Genomic Diversity in ICU-Associated *S. marcescens*

*S. marcescens* is a well-recognized opportunistic pathogen associated with HAIs, particularly in ICU settings where host immunity is frequently compromised [3,37,38]. It has been increasingly implicated in a wide spectrum of infections, including ventilator-associated pneumonia, bloodstream infections, urinary tract infections, and surgical site infections, reflecting its adaptability to diverse clinical niches [18,27]. In ICU populations, *S. marcescens* demonstrates a clear predilection for respiratory tract colonization and infection, with studies reporting that up to 86.59% of clinical isolates originate from respiratory samples [39]. This predominance is consistent with earlier findings indicating that tracheal aspirates and sputum represent the most common isolation sites, followed by blood and other sterile body fluids [37,39,40,41]. Such distribution patterns highlight the central role of mechanical ventilation and airway instrumentation in facilitating pathogen entry and colonization [39,42].

The clinical burden of *S. marcescens* infections in ICU patients is further exacerbated by host-related risk factors, including advanced age, underlying comorbidities, and exposure to invasive procedures [2,31,39]. Patients admitted to ICUs with *Serratia* infections are typically elderly, with a median age approaching 69 years, and show a male predominance of approximately 68.9% [2,27,43,44]. Key risk factors consistently identified include mechanical ventilation, tracheostomy, central venous catheterization, major surgery, and prolonged or high-dose antibiotic exposure [27,39,42,44,45].

Clinically, ICU-associated *S. marcescens* infections often present with fever, respiratory symptoms such as cough and dyspnea, and laboratory abnormalities including leukocytosis and neutrophilia [2]. Severe pneumonia occurs in approximately 16.7% to 28.6% of infected patients, underscoring the pathogen’s clinical significance in critically ill populations [2,3]. Mortality rates remain substantial, particularly in vulnerable cohorts such as burn patients, where overall mortality may reach 23%, and early infection is strongly associated with worse outcomes [38,42,43].

From an epidemiological perspective, *S. marcescens* is notable for its ability to cause nosocomial outbreaks, ranking as the fifth most common pathogen implicated in hospital infection clusters worldwide [46,47]. Outbreak investigations have demonstrated rapid transmission within ICUs, often occurring within days of admission, with a median time to bloodstream infection detection of 3 days (range: 1–6 days) and a mean time to culture positivity of 8.7 days [38,43]. Environmental reservoirs play a pivotal role in the persistence and dissemination of *S. marcescens* in ICU settings, with hospital water systems, sinks, and medical equipment serving as important sources of contamination [19,40].

Genomic analyses have confirmed clonal relationships between environmental and clinical isolates, supporting the hypothesis of environment-to-patient transmission pathways [40]. At the molecular level, *S. marcescens* exhibits remarkable genomic diversity, with multiple species within the *S. marcescens* complex coexisting in ICU environments [25]. Recent genomic studies have identified at least five distinct species within ICU-associated *Serratia* populations—*S. ureilytica*, *S. sarumanii*, *S. bockelmannii*, *S. marcescens*, and *S. nevei*—reflecting extensive evolutionary adaptation to heterogeneous hospital environments [2,25]. This diversity is driven by both core and accessory genome variations, enabling adaptation to environmental pressures such as antibiotic exposure and disinfectant use [25,48]. Horizontal gene transfer further contributes to the molecular epidemiology of *S. marcescens*, particularly in the dissemination of AMR determinants [25,48]. Advanced molecular typing techniques, including matrix-assisted laser desorption/ionization time-of-flight mass spectrometry (MALDI-TOF MS) and whole-genome sequencing (WGS), have become essential tools for outbreak detection and epidemiological surveillance [49,50]. These approaches enable accurate discrimination of clonal lineages and identification of transmission pathways, thereby supporting timely infection control interventions [49,50].

Finally, ICU epidemiology of *S. marcescens* is strongly influenced by healthcare practices, including antibiotic stewardship, infection control measures, and the use of personal protective equipment [45]. Inadequate adherence to infection prevention protocols, such as improper use of protective equipment or suboptimal hand hygiene, has been identified as a key driver of cross-transmission and outbreak amplification [45,51].

## 4. Molecular Ecology of Environmental Persistence and Device-Associated Transmission

### 4.1. Hospital Water Systems and Wet Surfaces

From a molecular ecological perspective, ICU water systems should be viewed not only as environmental reservoirs but also as selective niches that support biofilm formation, plasmid maintenance, stress-response activation, and persistence of genetically related *S. marcescens* lineages. *S. marcescens* exhibits marked environmental persistence in hospital settings because it can survive in moist ecological niches and colonize water-associated microenvironments for prolonged periods, particularly when biofilm formation occurs on plumbing-related surfaces [32,52,53,54,55]. Recent ICU-based genomic and ecological studies further indicate that hospital water systems are not merely transiently contaminated sites, but may function as stable environmental reservoirs that maintain *Serratia* populations over time and facilitate ongoing transmission opportunities [25,52].

In particular, sink drains, sink traps, aerators, and wet sink-adjacent surfaces appear to be among the most important persistence niches, because these sites support biofilm accumulation and repeated re-seeding of the surrounding environment [25,52,56,57]. A longitudinal genomic analysis from ICU settings showed that sink-associated *S. marcescens* complex populations could persist in the hospital environment as “source” reservoirs, whereas patients acted as more transient “sink” hosts, supporting a source–sink model of endemicity [25]. This framework suggests that environmental persistence is not solely due to repeated patient contamination, but also reflects the long-term ecological maintenance of clones and plasmids within the ICU water environment.

The persistence problem is further compounded by the ability of sink-associated reservoirs to harbor epidemiologically important resistance determinants, including carbapenemase-associated plasmids, thereby sustaining both organism survival and resistance-gene circulation within hospitals [56]. Evidence from neonatal and adult critical care units has repeatedly linked sink drains and sink traps with patient colonization or infection, indicating that wet plumbing structures are not passive contaminants but active reservoirs involved in transmission [32,52,53]. In a NICU study, all faucet-water and tap-aerator samples were negative for *S. marcescens*, whereas sink drains showed frequent positivity, with an average detection rate of 44%, strongly suggesting that the drain environment, rather than incoming tap water, was the dominant ecological niche [52]. The same study also found that some environmental and clinical strains remained detectable in sink drains for as long as one year after initial sampling, directly demonstrating prolonged persistence of clinically relevant strains within the plumbing environment [52].

Outbreak investigations also support a mechanistic role for splash and droplet dissemination from contaminated sinks [32,58,59,60]. One study reported that a bed-to-sink distance of <0.8 m was associated with markedly increased odds of colonization during a NICU outbreak, providing epidemiological support for the concept that splash-zone proximity influences transmission risk [32]. WGS in that outbreak linked contaminated handwashing sinks and patient isolates, further confirming that environmental persistence in sink structures can translate into clinically relevant cross-transmission [32]. Similarly, outbreak-associated clones have been identified in sink outlets and multiple sink traps within an ICU environment, and root-cause analysis revealed that relatively high tap-water use was a shared characteristic among affected cases [53]. These observations indicate that environmental persistence is strengthened not only by microbial biofilm ecology, but also by water-related clinical workflows that repeatedly connect contaminated plumbing systems with patient-care processes [40,53].

Contaminated hospital water environments, including sinks, continuous renal replacement therapy (CRRT) systems, and wastewater collection areas, have been shown to contribute to nosocomial outbreaks of carbapenemase-producing *S. marcescens*, indicating that environmental persistence can extend across interconnected wet infrastructure rather than remaining confined to a single fixture [40]. These findings further suggest that wastewater-handling practices and shared effluent systems may sustain or amplify reservoir continuity across multiple ICU areas, thereby facilitating ongoing transmission dynamics within healthcare settings [40].

Another clinically important aspect of environmental persistence is the contamination of liquid-associated products used near wet care areas [32,58]. Prior evidence indicates that *S. marcescens* outbreaks have been associated with a wide range of environmental and procedural reservoirs, including inadequately disinfected medical devices, water distribution systems, sinks, contaminated soap, medications, disinfectants, feeding tubes, breast pumps, breast milk, central venous catheters, and the hands of healthcare workers, underscoring the organism’s capacity to persist across both wet environments and patient-care interfaces [54,58,61,62,63]. In addition, aerosolization originating from sink-associated biofilms has been proposed as a mechanism for disseminating contamination to healthcare workers’ hands, adjacent surfaces, and milk-preparation items, highlighting how wet reservoirs can extend their influence beyond the immediate drainage site and seed secondary fomites within the clinical environment [58,59]. A second dominant transmission clone has also been identified circulating between liquid soap, sink surfaces, and healthcare workers’ hands, indicating that persistence within wet environments can extend into consumables and hand hygiene products, thereby bridging environmental and patient-care interfaces [32].

Accordingly, environmental persistence should be conceptualized as a network phenomenon involving plumbing-associated biofilms, splash-mediated dissemination, contaminated liquids, and human contact interfaces, rather than as contamination confined to a single isolated site [32,40,58]. Disinfection studies further demonstrate that once biofilms have matured, *S. marcescens* persistence becomes particularly difficult to eradicate, emphasizing the resilience of established environmental reservoirs [56]. Comparative evaluations of sink-drain interventions incorporating five approaches—self-disinfecting drains, chlorine disinfection, boiling-water treatment, hot-water flushing, and steam disinfection—have shown that chlorine-based strategies do not effectively reduce bacterial burden, whereas heat-based methods such as boiling water and steam achieve greater immediate reductions in both cultivable and intact cells [56,64,65,66,67]. However, even following apparently successful thermal interventions, *S. marcescens* clones can re-establish within drains containing mature and stable biofilms, indicating that biofilm-associated persistence is highly resilient and capable of rebound colonization despite control measures [56].

This finding is consistent with the broader observation that single decontamination procedures often fail to provide durable eradication of sink-associated opportunistic pathogens [53,56,68]. Repeated sink-disinfection attempts have been shown to provide only temporary clearance of bacteria from drainage systems, whereas sustained control requires a combination of environmental interventions and strict behavioral guidance regarding sink use, highlighting the importance of integrating engineering and human-factor approaches into infection-prevention strategies [53]. Multifaceted behavioral interventions combined with a transition to waterless patient care have been reported not to significantly reduce endemic ICU *S. marcescens* occurrence, suggesting that presumed water-related sources may not always represent the sole or dominant pathway of persistence [35]. These findings underscore the need to avoid oversimplifying the ecology of endemic *S. marcescens* as purely sink-driven; instead, persistence is likely multifactorial and may involve additional routes such as ventilation-related care practices, selective oral decontamination, and other underrecognized clinical reservoirs.

**Figure 2:**
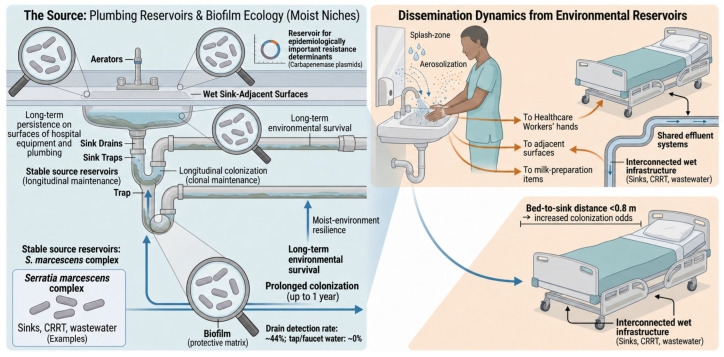
Environmental Reservoirs and Dissemination Pathways of *Serratia marcescens* in ICU Water-Associated Ecosystems. Created with BioRender.com (confirmation number: 6a326ec049311f6ebf2bbf9f).

Taken together, the currently available evidence supports the view that environmental persistence of *S. marcescens* in hospitals is driven by three interrelated mechanisms: long-term survival within moist biofilm-rich plumbing reservoirs, repeated dissemination from those reservoirs to adjacent fomites and hands, and incomplete eradication despite routine or intensified disinfection efforts [25,32,52,53,56]. These characteristics explain why *S. marcescens* can remain endemic in high-risk units even after apparent outbreak control, and why prevention strategies must address both microbial ecology and care-process design. Figure 2 summarizes the environmental reservoir–dissemination framework of *S. marcescens* in ICU water-associated ecosystems. It illustrates how plumbing reservoirs and biofilm-rich moist niches may sustain long-term persistence, while splash-mediated spread, healthcare workers’ hands, adjacent surfaces, liquid-associated items, and interconnected wet infrastructure may facilitate dissemination across ICU care environments.

### 4.2. Device- and Procedure-Associated Environmental Transmission: Ventilator Circuits and Contaminated Equipment

Recent evidence indicates that *S. marcescens* can persist and spread through device- and procedure-associated pathways in critical care and perioperative settings, making contaminated respiratory circuits, reusable equipment, and antiseptic solutions important reservoirs requiring focused infection-control attention [35,69,70,71]. In ICU settings, both invasive and non-invasive ventilation, particularly when combined with passive or active humidification systems, may facilitate the colonization of *S. marcescens* within respiratory circuits, although the relative contribution of each component remains incompletely defined [72]. Respiratory-circuit-associated acquisition can occur through exogenous routes, such as contamination during circuit maintenance and handling, as well as endogenous pathways, including aspiration of oropharyngeal secretions during procedures such as nebulization [35]. These findings highlight that both technical factors related to device management and patient-derived contamination play important roles in shaping transmission risk within ICU respiratory-care environments.

Measures proposed to reduce exogenous transmission include strict hand hygiene, minimization of circuit manipulation, use of closed tracheal suction systems, and placement of antimicrobial filters at inspiratory and expiratory circuit junctions, whereas endogenous transmission may be mitigated through bundled interventions such as subglottic suction, oral care, head-of-bed elevation, adequate cuff pressure, sedation monitoring, weaning assessment, and early mobilization [73]. An important limitation highlighted by the ICU investigation is that these potential lapses were not systematically evaluated, and neither colonized patients nor ventilator circuits underwent dedicated microbiological sampling, leaving the mechanistic chain of transmission only partially resolved [72]. Complementing this concern, full-length 16S rRNA sequencing of ICU ventilator circuits showed that bacterial community composition changed dynamically over time and that pathogenic taxa, including *S. marcescens*, became significantly more abundant by the third week of circuit use [69]. This finding suggests that prolonged circuit use may shift the circuit microbiome toward a more clinically hazardous state. Although that study emphasized overall pathogen succession rather than *S. marcescens* alone, the documented increase in *S. marcescens* during later sampling periods supports the view that this organism can participate in time-dependent circuit colonization under ICU conditions [69].

With the emergence of major disaster events such as COVID-19, shared ventilator strategies have been revisited and explored at both theoretical and experimental levels under conditions of extreme resource constraints. In this context, experimental studies provide a more mechanistic perspective: when heat and moisture exchanger (HME) filters were placed proximally, no cross-transmission of *S. marcescens* was detected across parallel circuit branches during a 24-h observation period, suggesting that appropriate filtration configurations can effectively reduce short-term cross-contamination under controlled conditions [74,75]. Follow-up trials in similarly structured co-ventilation systems further indicated that a single proximal HME filter may provide sufficient microbial barrier protection and that additional ventilator-side bacterial/viral filters may not always be necessary under the tested conditions [75,76]. However, these findings are limited to short-duration, highly controlled experimental settings, and their generalizability to prolonged or real-world clinical applications remains uncertain [75,76]. Therefore, these co-ventilation studies do not eliminate broader concerns regarding routine ICU ventilator colonization, as they assessed short-term controlled transmission rather than long-term circuit use, humidifier contamination, or repeated disconnections during clinical care [35,74,75].

Beyond respiratory equipment, outbreak investigations show that *S. marcescens* can also persist in complex reusable medical devices that contain hidden spaces or are subject to imperfect cleaning and disinfection workflows [70]. In a surgical-site outbreak, a T-shaped intraoperative ultrasound probe was eventually confirmed as the outbreak source after initial environmental cultures were negative; contamination was recovered only after improved sampling methods were used, including sterile-water immersion or injection into difficult-to-access probe recesses [70]. This investigation revealed key process failures, including the absence of a disposable sterile probe cover during intraoperative use and failure to perform final disinfection after cleaning, demonstrating how minor deviations in reprocessing practice can sustain clinically significant *S. marcescens* transmission.

A related but distinct transmission pattern was observed in an adult cardiac surgery outbreak, in which contaminated 2% aqueous chlorhexidine solution was identified as the source of postoperative *S. marcescens* mediastinitis and wound colonization [71]. That outbreak involved rapid postoperative presentation, frequent asymptomatic wound colonization, and microbiological concordance between unopened chlorhexidine bottles and patient isolates, indicating that antiseptic solutions may function as direct inoculation vehicles rather than protective barriers when manufacturing or sterility control fails [71]. The same report noted that *S. marcescens* contamination of aqueous chlorhexidine has been repeatedly implicated in medical and surgical ward outbreaks and that earlier studies had shown the organism’s ability to survive in chlorhexidine solutions for prolonged periods, whereas alcohol-based formulations appear less vulnerable to such contamination [71,77,78,79].

**Figure 3:**
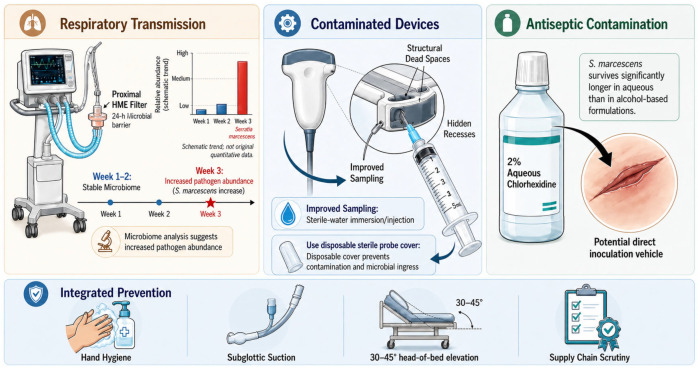
Device- and Procedure-Associated Transmission Pathways and Prevention Strategies for *Serratia marcescens* in Critical Settings. Created with BioRender.com (confirmation number: 6a326f5749311f6ebf2d6ada).

Taken together, these reports indicate that *S. marcescens* should not be viewed merely as a transient contaminant, because it can persist in humidified respiratory environments, reusable equipment with structural dead spaces, and even disinfectant products intended for infection prevention [35,69,70,71]. From a practical ICU perspective, the available evidence supports a layered prevention approach that combines device-design awareness, strict circuit-handling protocols, standardized filter practices, surveillance of prolonged ventilator use, meticulous reprocessing of reusable equipment, and careful scrutiny of antiseptic products and supply chains [35,69,70,71]. To synthesize the proposed transmission mechanisms and corresponding prevention strategies, we summarized the device- and procedure-associated environmental pathways of *S. marcescens* transmission in Figure 3.

## 5. Genomic Surveillance, Molecular Typing, and Outbreak Containment

Timely identification of infection sources, transmission pathways, and clonal relationships is fundamental for controlling the nosocomial spread of *S. marcescens*, and molecular typing tools have become essential components of outbreak investigation and infection-control decision-making [20,31,80,81,82,83].

For *S. marcescens*, established multilocus sequence typing (MLST) schemes and higher-resolution WGS have been demonstrated to effectively discriminate strain relatedness, reconstruct transmission chains, and support epidemiological surveillance. However, conventional MLST and WGS remain constrained by cost, turnaround time, and labor intensity, prompting increasing interest in more rapid and accessible molecular approaches for practical outbreak control [31]. The molecular value of WGS extends beyond outbreak confirmation. By resolving single-nucleotide variation, accessory genome content, plasmid structures, resistance determinants, and strain-level relatedness, WGS provides a mechanistic framework for distinguishing recurrent environmental persistence from repeated introductions. This is particularly relevant for *S. marcescens*, in which clonal spread, plasmid-associated AMR, and biofilm-associated reservoirs may coexist within the same ICU ecosystem.

Recent studies further indicate that third-generation long-read nanopore sequencing enables rapid clonal identification during early outbreak stages. Although its higher base-calling error rate may limit single-nucleotide resolution, this approach offers important advantages for decentralized laboratories and time-sensitive infection-control settings [84,85,86,87]. Similarly, polymerase chain reaction (PCR)-based strategies have been shown to be effective tools for rapid detection of colonized patients during outbreaks, bridging the gap between conventional culture and WGS by shortening diagnostic turnaround time, accelerating isolation decisions, and facilitating early interruption of transmission chains [81,87].

Beyond molecular tools, the literature consistently emphasizes that active surveillance combined with sustained non-pharmacological infection-control measures constitutes the foundation of *S. marcescens* outbreak containment [7,23,88,89]. A longitudinal surveillance study demonstrated that although major outbreaks can be rapidly controlled through early implementation of hand hygiene, contact isolation, patient cohorting, and dedicated staffing, smaller clusters may persist over extended periods after the initial outbreak has resolved. This finding highlights the necessity of maintaining surveillance and infection-control bundles even when no new cases are temporarily detected [7].

Comprehensive intervention packages, including outbreak management teams, frequent multidisciplinary meetings, twice-weekly active screening, environmental sampling, reinforced contact precautions, establishment of satellite units, and education of healthcare workers and families, have been shown to effectively reduce transmission in neonatal intensive care settings [88]. Consistent findings from earlier studies also support that strict hand hygiene, enhanced environmental cleaning, active patient screening, contact isolation, and health education are sufficient to rapidly control *S. marcescens* outbreaks in NICUs [89]. Rapid and comprehensive responses initiated from the first detected case, including intensified screening frequency, multidisciplinary coordination, spatial isolation, dedicated nursing assignments, environmental source investigation, and molecular characterization, are critical to preventing further spread [23].

Among all interventions, hand hygiene remains the most consistently validated and impactful control measure [7,39,90]. Long-term surveillance combined with WGS and phylogenetic analysis has demonstrated that post-outbreak clusters are often genetically unrelated to the initial outbreak strain, and the marked reduction in incidence following intervention supports the effectiveness of implemented infection-control measures [7]. In addition, the absence of *S. marcescens* in healthcare workers’ hand cultures suggests that transient contamination, rather than persistent colonization, may represent the primary transmission route [7].

Field investigations have further identified deficiencies in hand hygiene, unsafe injection practices, improper disinfection of medical devices, inadequate storage of ultrasound gel, and suboptimal environmental cleaning as key contributors to outbreak occurrence. Targeted training with continuous monitoring and feedback has been shown to significantly improve compliance and eliminate new cases [27,90]. From an AMR perspective, inconsistent infection-control practices and inappropriate antibiotic use may jointly drive the emergence and dissemination of resistant strains, underscoring the need for integrated strategies combining antimicrobial stewardship, surveillance, hand hygiene training, and rapid response to clustered cases [39,91].

Another critical aspect highlighted in recent literature is the role of moist environments and water-associated reservoirs in *S. marcescens* transmission [7,32,43,92]. Evidence from adult ICU outbreaks suggests that disinfectant-resistant strains may spread from sink areas to patient zones via cleaning equipment, subsequently contaminating surfaces and being transmitted through healthcare workers’ hands [32,43]. Moreover, *S. marcescens* has been shown to persist in water systems, surfaces, and plumbing infrastructure, and may remain detectable even after disinfection [32,43].

Systematic environmental screening has demonstrated that positive drain sites correlate with case distribution, and removal of sinks combined with water-free care strategies can effectively prevent outbreak recurrence [92]. Additional environmental sources, including medical devices, medications, soap dispensers, and milk products, have also been implicated, highlighting the need to extend infection-control strategies beyond direct patient contact to encompass environmental and fluid-related transmission pathways [7,93,94,95,96].

Active screening strategies and patient cohorting are also essential components of outbreak containment [23,32,88,92]. Recent studies have incorporated staffing patterns and workload into outbreak risk analysis frameworks [32,90,92]. Increased rotation of nursing staff has been associated with higher infection or colonization risk, likely due to expanded contact networks and reduced adherence to infection-control practices under suboptimal nurse-to-patient ratios [32,97,98]. High bed occupancy and workload pressure may further compromise hygiene compliance and interact with strain-specific characteristics to facilitate transmission within NICU environments [92]. Field investigations also demonstrate that inadequate staffing and mismatched cleaning workflows represent structural vulnerabilities that can be directly targeted during outbreak response [90].

Collectively, recent evidence indicates that infection control for *S. marcescens* has evolved from traditional hand hygiene and isolation approaches to integrated strategies incorporating genomic surveillance, active screening, environmental reservoir management, patient cohorting, continuous education, and antimicrobial stewardship [7,31,32,43,84,87,88,92]. Key conclusions from recent studies include that active screening is essential for capturing hidden transmission beyond clinical cases, water systems and moist reservoirs represent persistent sources of contamination, hand hygiene remains the most impactful intervention, rapid molecular tools shorten outbreak detection and response time, and staffing patterns and workflow constraints significantly influence transmission dynamics [7,31,32,43,84,87,88,92].

Future research should focus on developing scalable screening algorithms for ICU and NICU settings, evaluating the cost-effectiveness of rapid molecular diagnostics, clarifying the relative contribution of environmental reservoirs across healthcare environments, assessing long-term outcomes of water-free care and infrastructure redesign, and integrating genomic, epidemiological, and antimicrobial-use data to build predictive models for *S. marcescens* transmission [3,39,84,87,92].

## 6. Biofilm-Mediated Persistence, Antimicrobial Tolerance, and Emerging Anti-Biofilm Strategies in *Serratia marcescens*

Biofilm formation represents a critical virulence and survival strategy of *S. marcescens*, enabling persistent colonization on both biotic and abiotic surfaces and contributing to immune evasion and antimicrobial tolerance in ICU settings [3,99,100,101]. Biofilms consist of structured microbial communities embedded in an extracellular polymeric substance (EPS) matrix composed of polysaccharides, proteins, lipids, and extracellular DNA (eDNA), which enhances structural stability, nutrient retention, and horizontal gene transfer [3,100,102].

Initial adhesion is mediated by type 1 fimbriae, flagella, and outer membrane proteins, and is tightly regulated by the cAMP–CRP system in response to environmental nutrient availability [99,103,104]. Additional regulatory mechanisms involve redox-sensitive transcription factors such as OxyR and intracellular cAMP signaling pathways, which dynamically modulate fimbrial expression and early-stage biofilm formation [104,105]. Quorum sensing (QS) has long been studied primarily through the *SpnI*/*SpnR* system in *S. marcescens* [106,107]. However, recent evidence suggests that N-acyl homoserine lactone (AHL)-mediated regulatory networks, particularly the *swrI*/*swrR* and *SmaI*/*SmaR* systems, play central roles in coordinating biofilm maturation, EPS production, motility, enzyme secretion, and prodigiosin biosynthesis [108]. Disruption of QS signaling has been shown to significantly reduce biofilm biomass and virulence gene expression, highlighting QS as a key therapeutic target [109,110,111,112]. Mechanistically, QS inhibition using 3-phenylpropan-1-amine resulted in a 48% reduction in biofilm formation and downregulation of key genes, including *fimA*, *fimC*, *bsmB*, *flhC*, and *flhD*, as demonstrated by quantitative reverse transcription polymerase chain reaction (qRT-PCR) and imaging analyses [112].

Recent findings further indicate that extracellular proteolysis contributes to biofilm maturation, with the metalloprotease PrtA facilitating flagellar turnover and structural development of mature biofilms [113]. Biofilm-associated AMR is multifactorial and includes limited antibiotic penetration, altered metabolic states such as persister cell formation, and upregulation of efflux pumps and stress-response pathways [3,39,114,115]. Efflux pump systems, particularly ATP-binding cassette (ABC) transporters, play a major role in resistance, where adenosine triphosphate (ATP) hydrolysis drives conformational changes that export antimicrobial substrates across membranes [6,116,117]. Specific ABC transporters such as SmdAB and MacAB have been associated with multidrug resistance, biofilm formation, and survival under oxidative stress conditions, further linking efflux systems to virulence and persistence [6,118].

Proteomic studies under carbapenem stress have demonstrated increased expression of biofilm-associated proteins such as OmpA, ABC transporter ATP-binding proteins, LptB, Lpp, and MreB, suggesting adaptive remodeling of membrane integrity and efflux activity during antimicrobial exposure [37]. Clinical data support a strong association between biofilm formation and AMR, with studies reporting that all examined *S. marcescens* isolates formed biofilms and that higher extended-spectrum β-lactamase (ESBL) gene prevalence correlated with stronger biofilm-forming capacity [119,120,121].

Environmental persistence is also closely linked to biofilm formation, as *S. marcescens* has been frequently identified within biofilms in sink drains, aerators, and hospital water systems, serving as reservoirs for ICU outbreaks [35,52]. Biofilm-associated cells may enter viable-but-non-culturable (VBNC) states under environmental stress, limiting detection by conventional culture methods and necessitating molecular or flow cytometry-based monitoring approaches [56,122]. Inadequate disinfection practices, such as insufficient contact time of quaternary ammonium compounds, may induce stress responses that enhance efflux activity, membrane remodeling, and biofilm formation, thereby increasing environmental tolerance [43,123,124,125].

Emerging therapeutic strategies targeting biofilms include bacteriophage therapy, phage–antibiotic synergy, QS inhibitors, type VI secretion system (T6SS)-targeted antivirulence agents, and nanoparticle-based drug delivery systems [3]. Phage–antibiotic synergy has demonstrated enhanced biofilm disruption and delayed resistance development when combined with sub-inhibitory antibiotic concentrations, particularly in device-associated infections [126]. Overall, recent evidence suggests that biofilm formation in *S. marcescens* is a highly integrated process involving adhesion, QS, proteolysis, efflux-mediated resistance, and environmental adaptation, and should be considered a central driver of ICU-associated persistence and treatment failure [3,37,56,121]. The major biofilm-associated determinants contributing to persistence, antimicrobial tolerance, and resistance in *S. marcescens* are summarized in Table 1.

## 7. Integrated Discussion and Future Perspectives

*S. marcescens* should be understood as a highly ICU-adapted opportunistic pathogen rather than a purely episodic outbreak organism, because recent studies demonstrate its strong capacity to persist and adapt across hospital water systems, respiratory equipment, wet surfaces, medical devices, and patient-associated reservoirs [6,7,40]. The ICU environment creates a high-risk ecological interface in which critically ill patients, invasive devices, antimicrobial exposure, environmental reservoirs, and biofilm-associated niches converge to facilitate colonization, infection, and recurrent transmission [7,31].

Water-associated reservoirs remain central to ICU transmission, as contaminated sinks, drainage systems, wet surfaces, and wastewater-related sites have repeatedly been implicated in *S. marcescens* acquisition and outbreak persistence [40,53]. Respiratory care pathways should also be incorporated into future *S. marcescens* prevention strategies because ICU ventilator circuits, humidification interfaces, nebulizer use, circuit manipulation, and respiratory tract colonization may provide acquisition routes beyond hospital water reservoirs alone [7,39].

Experimental co-ventilation studies performed under disaster-medicine conditions showed that proximal HME filtration prevented detectable short-term cross-transmission of *S. marcescens* across parallel circuit branches, and subsequent work suggested that a single proximal HME filter may provide sufficient microbial barrier protection under controlled experimental conditions [74,75,76]. Nevertheless, these findings remain limited to short-duration laboratory settings and should not be extrapolated to routine ICU ventilation, where prolonged circuit use, humidifier contamination, repeated disconnections, nebulizer exposure, and airway-care practices may sustain opportunities for colonization and transmission [35,74,75]. Accordingly, ventilator-associated care bundles, respiratory equipment auditing, airway management protocols, and filtration-configuration assessment should be integrated into broader *S. marcescens* infection-control frameworks.

Biofilm-associated persistence provides a plausible explanation for the difficulty of eradicating *S. marcescens* from hospital environments, because embedded bacterial communities can tolerate disinfectants, survive in water-associated niches, and contribute to recurrent contamination [6,7]. AMR further complicates ICU management, as *S. marcescens* possesses intrinsic resistance mechanisms, inducible AmpC β-lactamase activity, efflux pumps, and the ability to acquire plasmid-mediated resistance determinants [84,127]. Antimicrobial stewardship should therefore be considered part of outbreak prevention rather than only treatment optimization, because hospital-level antibiotic use has been associated with changes in *S. marcescens* resistance patterns and may select for resistant subpopulations in ICU ecosystems [39,40].

Genomic surveillance has become essential for distinguishing clonal outbreaks from polyclonal endemicity, confirming epidemiological links between clinical and environmental isolates, and detecting prolonged colonization before overt infection [84,88]. Species-level misclassification within the *S. marcescens* complex also indicates that conventional MALDI-TOF MS may not adequately resolve clinically relevant genomic diversity, making WGS or other high-resolution methods important for future ICU epidemiology [127]. Rapid nanopore sequencing offers a practical direction for decentralized outbreak response, because it can provide near-real-time genomic information while remaining more accessible than conventional high-throughput sequencing platforms [84].

Future infection-control strategies should move from isolated interventions toward integrated bundles that combine hand hygiene, patient cohorting, environmental decontamination, sink and drainage management, ventilator-circuit auditing, active surveillance, and antimicrobial stewardship [7,88]. Research on anti-biofilm strategies, environmental engineering, precision disinfection, and reservoir-specific sampling protocols is needed to address persistent contamination that may not be eliminated by routine cleaning or single structural interventions [7,53]. An integrated future model for *S. marcescens* prevention in ICUs should combine molecular epidemiology, environmental microbiology, respiratory-care practices, antimicrobial stewardship, anti-biofilm strategies, and emerging artificial intelligence (AI)-assisted surveillance approaches within an adaptive infection-control framework [7,84,88,128]. Therefore, future research should prioritize prospective multicenter surveillance, standardized outbreak definitions, harmonized environmental sampling protocols, integration of genomic and metagenomic approaches, and clinically oriented studies linking biofilm biology, AMR, and patient outcomes in ICU populations.

## 8. Limitations

Several limitations should be considered when interpreting the findings of this integrative review. First, the current evidence base on *S. marcescens* in ICU settings is largely derived from outbreak reports, retrospective observational studies, and single-center investigations, which may limit the generalizability of conclusions across different healthcare systems, ICU infrastructures, and infection-control practices [46,47,51]. In addition, a considerable proportion of published data originates from NICU outbreaks, and the transmission dynamics, host vulnerability, antimicrobial exposure patterns, and clinical outcomes observed in NICU populations may not be directly applicable to adult critically ill patients [9,20,22,23,88]. Substantial heterogeneity across studies, including differences in microbiological identification methods, molecular typing approaches, surveillance intensity, environmental sampling protocols, and infection-control interventions, further restricts direct comparison and weakens the strength of cross-study synthesis [80,81,84,86,88].

Another important limitation is that many investigations rely primarily on culture-based detection methods, which may underestimate the true burden of colonization and fail to identify low-level or subclinical transmission, particularly in biofilm-associated reservoirs and intermittently contaminated environments. Environmental sampling strategies also vary widely among studies, and intermittent recovery of *S. marcescens* from sinks, drains, water systems, medical devices, and wet surfaces suggests that conventional surveillance may not fully capture persistent environmental reservoirs or transmission pathways [52,53,56,122]. Moreover, the association between antimicrobial exposure and resistance emergence has not been systematically assessed in many reports, making it difficult to establish causal relationships between antibiotic selection pressure, outbreak persistence, and the evolution of multidrug-resistant *S. marcescens* strains [39,40]. Inconsistent definitions of colonization, infection, and outbreak thresholds across institutions may also influence reported incidence, clinical outcomes, and the perceived effectiveness of infection-control measures.

Finally, although biofilm formation is increasingly recognized as a key factor contributing to environmental persistence, device-associated contamination, and antimicrobial tolerance in *S. marcescens*, most mechanistic evidence remains derived from in vitro experiments or theoretical models rather than ICU-based translational studies [99,103,104,105].

## 9. Conclusions

*S. marcescens* has evolved from a traditionally underrecognized environmental organism into a clinically relevant ICU-associated pathogen characterized by substantial ecological adaptability, genomic diversity, biofilm-mediated persistence, and complex AMR mechanisms. Evidence from the past decade indicates that ICU transmission is sustained by the convergence of vulnerable critically ill hosts, invasive devices, antimicrobial selection pressure, hospital water systems, wet-surface reservoirs, ventilator-related equipment, and contaminated liquid-associated products. At the molecular level, inducible AmpC β-lactamase activity, efflux-mediated tolerance, plasmid-associated resistance transfer, QS regulation, and biofilm-associated survival collectively contribute to environmental persistence, therapeutic difficulty, and recurrent outbreak potential. These findings suggest that effective control of *S. marcescens* in ICUs requires an integrated framework combining active surveillance, genomic tracking, antimicrobial stewardship, environmental reservoir management, respiratory-care auditing, device reprocessing, and targeted anti-biofilm strategies. Future studies should prioritize prospective multicenter surveillance, standardized environmental sampling, high-resolution genomic and metagenomic approaches, and translational investigations linking biofilm biology, AMR, and patient outcomes. Overall, *S. marcescens* should be regarded not merely as an episodic outbreak organism, but as an adaptable ICU-associated pathogen requiring sustained, multidisciplinary, and ecology-informed infection-control strategies.

## Figures and Tables

**Table 1:** Biofilm-Associated Determinants Involved in Persistence, Antimicrobial Tolerance, and Resistance in *Serratia marcescens*.

Determinant	Description Reported in This Review	Contribution to Persistence and Antimicrobial Tolerance
EPS matrix	Structural matrix composed of polysaccharides, proteins, lipids, and eDNA	Enhances structural stability, nutrient retention, and horizontal gene transfer.
Type 1 fimbriae	Initial adhesion factor	Contribute to initial adhesion and early-stage biofilm formation.
Flagella	Initial adhesion factor	Contribute to initial adhesion and early-stage biofilm formation.
cAMP–CRP system	Regulatory system responsive to nutrient availability	Regulates early-stage biofilm formation.
OxyR	Redox-sensitive transcription factor	Dynamically modulates fimbrial expression and early-stage biofilm formation.
*SpnI*/*SpnR* quorum- sensing system	QS regulatory system	Participates in QS-mediated biofilm regulation.
*swrI*/*swrR* and *SmaI*/*SmaR* systems	AHL-mediated regulatory network	Coordinate biofilm maturation and biofilm-associated functions.
PrtA metalloprotease	Extracellular protease	Contributes to biofilm maturation.
ABC transporters	ATP-dependent efflux transporters	Contribute to resistance during antimicrobial exposure.
SmdAB and MacAB transporters	ABC transporter family members	Link efflux systems to virulence and persistence.
OmpA, LptB, Lpp, and MreB	Biofilm-associated proteins upregulated during carbapenem stress	Suggest adaptive remodeling of membrane integrity and efflux activity during antimicrobial exposure.

Abbreviations: ABC, ATP-binding cassette; AHL, N-acyl homoserine lactone; ATP, adenosine triphosphate; CRP, cyclic AMP receptor protein; eDNA, extracellular DNA; EPS, extracellular polymeric substance; QS, quorum sensing.

## Data Availability

No new data were created or analyzed in this study. Data sharing is not applicable to this article.

## Abbreviations

The following abbreviations are used in this manuscript:

ABC	ATP-binding cassette
AHL	N-acyl homoserine lactone
AI	artificial intelligence
AMR	antimicrobial resistance
ATP	adenosine triphosphate
COVID-19	coronavirus disease 2019
CRE	carbapenem-resistant Enterobacterales
CRRT	continuous renal replacement therapy
eDNA	extracellular DNA
EPS	extracellular polymeric substance
ESBL	extended-spectrum β-lactamase
HAIs	healthcare-associated infections
HME	heat and moisture exchanger
ICU	intensive care unit
ICUs	intensive care units
MALDI-TOF MS	matrix-assisted laser desorption/ionization time-of-flight mass spectrometry
MLST	multilocus sequence typing
NICU	neonatal intensive care unit
NICUs	neonatal intensive care units
PCR	polymerase chain reaction
PRISMA	Preferred Reporting Items for Systematic Reviews and Meta-Analyses
qRT-PCR	quantitative reverse transcription polymerase chain reaction
QS	quorum sensing
*S. marcescens*	*Serratia marcescens*
T6SS	type VI secretion system
VBNC	viable-but-non-culturable
WGS	whole-genome sequencing
